# Vaginal Cuff Dehiscence: Two Case Reports and a Review of the Literature

**DOI:** 10.3390/jcm12134187

**Published:** 2023-06-21

**Authors:** Babette Jaime Moens, Antonino Buonomo, Philippe De Sutter

**Affiliations:** 1 Department of Gynaecology, Universitair Ziekenhuis Brussel, Laarbeeklaan 101, 1090 Jette, Belgium; philippe.desutter@uzbrussel.be; 2 Department of Gynaecology, Centre Hospitalier Universitaire Tivoli, Avenue Max Buset 34, 7100 La Louviere, Belgium; antonino.buonomo@chu-tivoli.be

**Keywords:** vaginal dehiscence, hysterectomy complication, vaginal cuff closure, laparoscopy, robotic hysterectomy, evisceration, risk factors

## Abstract

Vaginal cuff dehiscence (VCD) is a rare but serious condition associated with high morbidity, especially in the presence of an evisceration. It usually occurs as a complication of hysterectomy, but has also been reported after other pelvic surgeries. In this article, we will present two cases of vaginal cuff dehiscence with evisceration in post-menopausal patients. Both cases occurred post-operatively, the first after a laparoscopic radical hysterectomy and the other after a trachelectomy performed by robotic-assisted laparoscopy (with a prior history of subtotal hysterectomy). Both cases were treated surgically, the first by a combined laparoscopic and vaginal approach, and the second case only by laparoscopic approach. The main risk and protective factors are discussed in a narrative literature review which summarizes the available evidence on this rare condition, discussed by type of study designs and thus evidence level. A laparoscopic vaginal cuff closure is the most protective factor in preventing VCD, compared to a vaginal closure. Clinicians should be aware of this condition and of its risk factors and precipitating events in order to identify high-risk patients. Knowledge of these allows prompt recognition, which is crucial for adequate management, for which multiple approaches have been described.

## 1. Introduction

Vaginal cuff dehiscence (VCD), is defined as the partial or complete separation of the vaginal vault, and may be associated with an evisceration. It is a complication of total hysterectomy or other pelvic surgery, with the first known case published by Hobbs in 1952 [1]. In 1994, in an article where they reported three cases of VCD, Somkuti et al. [2] described several risk factors that could contribute to the weakening of the vaginal apex after vaginal or abdominal surgery: (1) poor surgical techniques, (2) postoperative wound or cuff infection, (3) wound hematoma, (4) resumption of sexual activity before complete healing, (5) advanced age, (6) previous radiation therapy, (7) chronic steroid administration that prevents adequate healing, (8) trauma and rape, (9) previous vaginoplasty, and (10) Valsalva’s maneuver after a vaginal hysterectomy or straining during a bowel movement. To this day, some of these risk factors are still considered accurate. Knowledge of possible risk factors for VCD is important, especially since there might be a rise in VCD incidence over the years, parallel to the rise of laparoscopic hysterectomies [3].

In this article, we will present two cases of vaginal cuff dehiscence and evisceration following hysterectomy and trachelectomy (with prior hysterectomy) by laparoscopy and robot-assisted laparoscopy, respectively. A literature review was performed to identify possible risk factors and their importance in this condition.

## 2. Case Presentations

### 2.1. Case 1

A 69-year-old woman presented to the emergency room in January 2019 because of protruding bowels from her vagina, accompanied by important hypogastric pain, after she tried defecating.

She had a medical history of three vaginal births, and a laparoscopic radical hysterectomy (Wertheim procedure) with pelvic lymphadenectomy for a cervix carcinoma (staged FIGO IB1) performed 1 week earlier in another hospital. No complications during the surgery were reported. She had no prior surgical history.

Upon arrival at the hospital, a large section of small bowel was protruding out of the vagina. The patient was brought immediately to the operating theater, where the intestinal loops were repositioned vaginally under general anesthesia. A simultaneous laparoscopy was performed, confirming the viable aspect of the intestines. The remaining V-loc^®^ thread at the vaginal vault seemed to have ruptured in the middle of the suture. The residual V-loc^®^ was removed transvaginally. The vaginal vault was then closed by transvaginal, separate X-sutures with Vicryl^®^ 0. The bladder integrity was controlled by filling it with methylene blue, and showed no signs of leakage. Amoxicillin-clavulanic acid IV was started per-operatively and continued in the next few days. Despite the continuation of the antibiotic treatment, the post-operative recovery was complicated by signs of infections. A CT-scan showed the presence of an abscess, which was punctured. The IV antibiotic treatment was then switched to piperacillin/tazobactam. The patient was discharged on postoperative day 14. The patient received the necessary adjuvant radiotherapy + brachytherapy treatment. Follow-up until 2023 showed no signs of recurrence of the vaginal vault dehiscence.

### 2.2. Case 2

A 52-year-old patient presented herself at the emergency room in January 2022, with an intense hypogastric pain and a bulging sensation from the vagina. The pain started gradually after having consensual sexual intercourse on the evening before, but intensified after urinating in the morning.

She had a medical history of two vaginal births, one cesarean section, and a subtotal laparoscopic hysterectomy with intra-abdominal morcellation of the uterus in April 2014 (for the presence of a large myoma). In November 2021, because of persistent pelvic pain and the presence of peritoneal and ovarian endometriosis and morcellomas (confirmed by MRI), a trachelectomy with bilateral salpingo-oophorectomy, adhesiolysis and resection of endometriosis and morcellomas was performed by robot-assisted laparoscopy. The vaginal suture was performed endoscopic robotically using V-Loc^®^ 3.0 barbed suture. There were no immediate postoperative complications. The patient did not smoke, and did not have any other known disease that could compromise a normal tissue healing.

**Figure 1:**
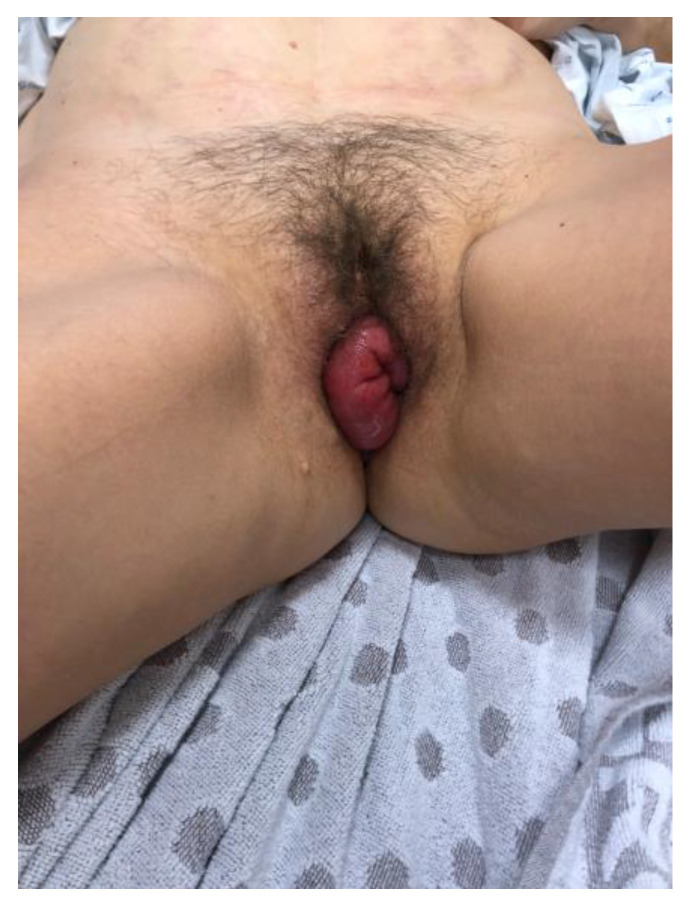
Evisceration of ileum through the vaginal cuff dehiscence.

Upon examination in the emergency room (10 weeks since her last surgery), an evisceration of the ileum through the vagina was noticed (Figure 1), thus a vaginal cuff dehiscence was suspected. The loops had a normal coloration and showed no signs of necrosis. The vulva and eviscerated content were rinsed using a chlorhexidine digluconate solution, and were then delicately restituted into the abdominal cavity. The vagina was then packed.

The patient was prepared for the operating theater and the surgery was performed within a 2 h delay (from the first moment of arrival to the emergency room). A complete blood work and a COVID test were collected, which were normal.

**Figure 2:**
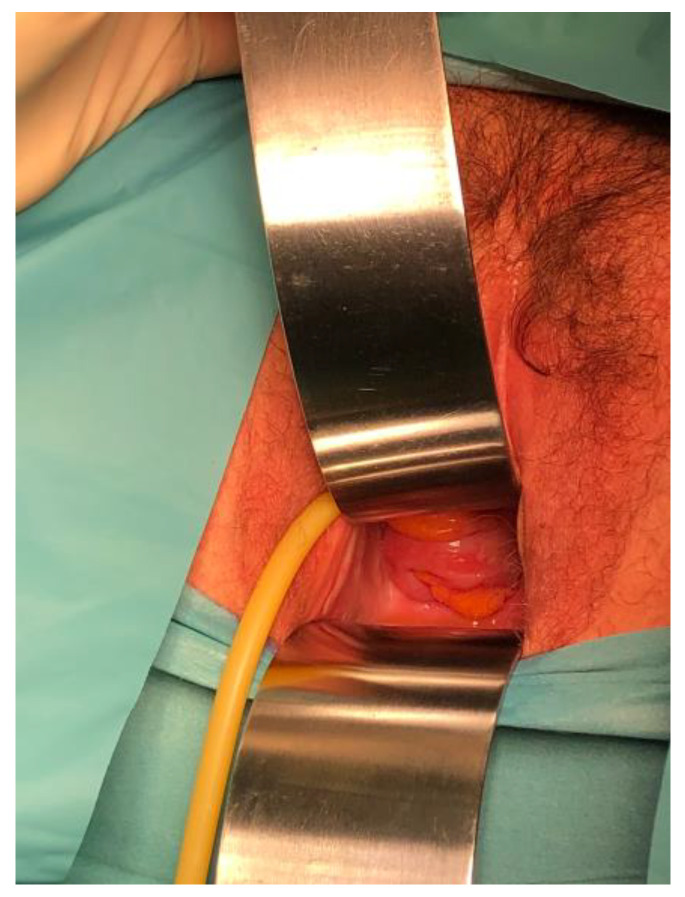
Visualization of the sigmoid through the vaginal cuff dehiscence.

An exploratory laparoscopy was performed after the placement of a urinary catheter, exploration (Figure 2) and then packing of the vagina. The patient received 2 g of Cefazolin one-shot as surgical prophylaxis. Given her surgical history, the insufflation and first trocar were placed in Palmer’s point.

**Figure 3:**
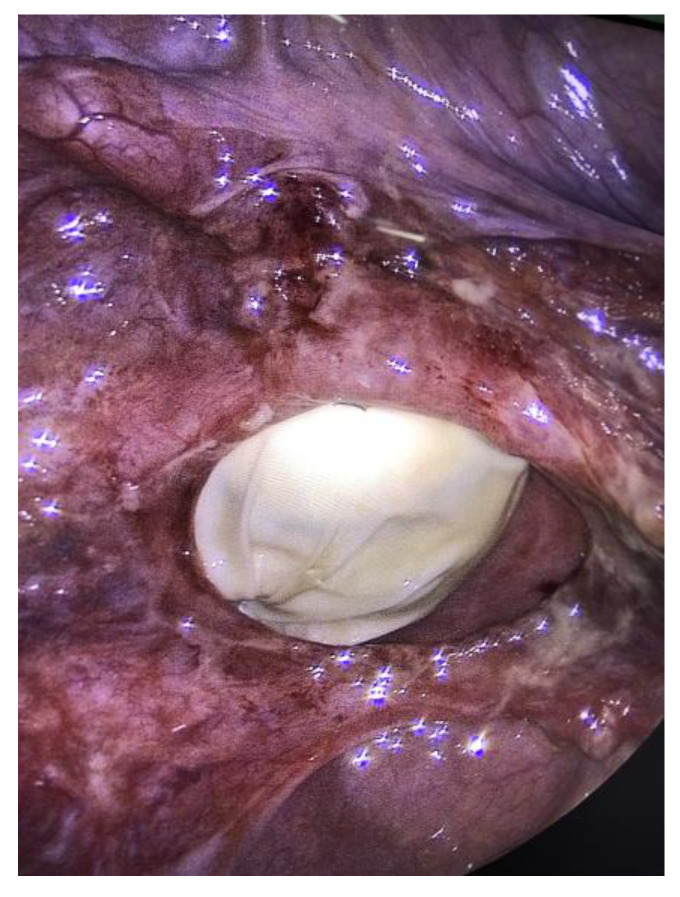
Laparoscopic view of the dehiscence, vagina packed with glove.

**Figure 4:**
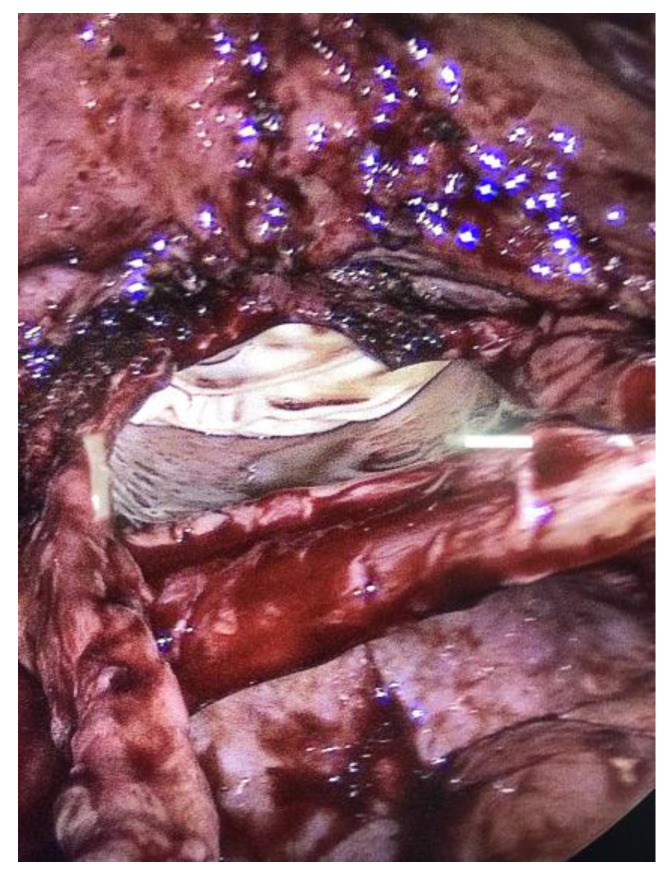
Trimming of the posterior vaginal edge.

**Figure 5:**
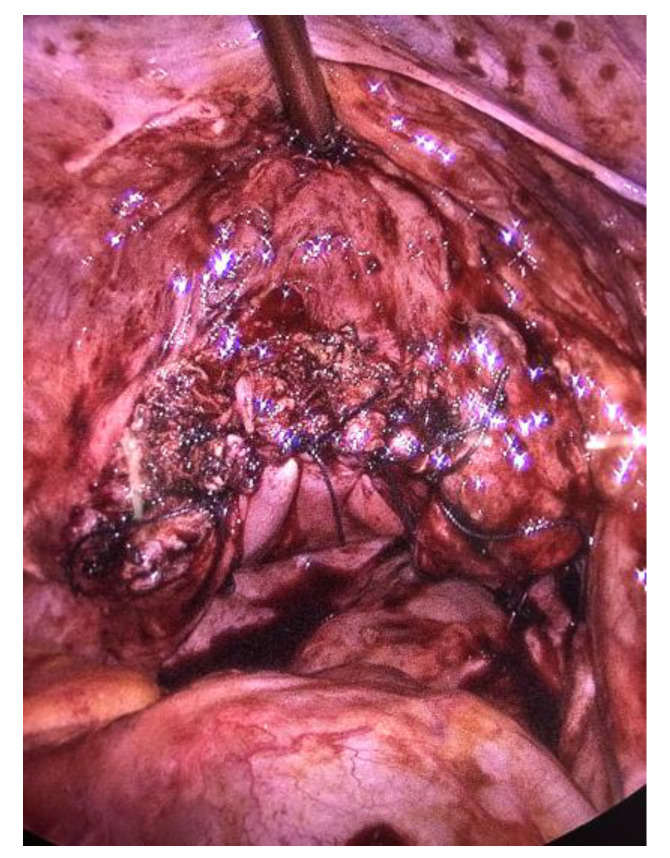
Vaginal cuff closed with X-sutures, filled bladder.

There were no signs of intestinal necrosis, and the vaginal cuff appeared open with signs of fibrosis on the edge (Figure 3). The bladder was filled with methylene blue to allow a clear definition of the bladder limits and a careful dissection was executed. The vaginal cuff edges were trimmed until healthy tissue was seen (Figure 4), then closed by four individual X sutures performed laparoscopically, using Vicryl 1^®^ braided resorbable thread (Figure 5). An intra-abdominal drain was placed.

Post-operatively, no complications were found; the drain was removed the second postoperative day and the patient returned home. An antibiotic treatment of seven days with amoxicillin-clavulanic acid was administered.

Peritoneal liquid taken during the surgery showed no signs of infection. The anatomopathological report of the trimmed vaginal cuff edges showed only signs of inflammatory granulation.

A hormonal substitution therapy (by transdermal estradiol 0.75 mg daily) was started three weeks postoperatively, since the patient presented with climacteric symptoms, and because of the potential benefit of substitution therapy on vaginal wound healing.

The consultations at 3, 6 and 12 weeks post-operatively were all normal and showed no signs of dehiscence.

## 3. Method

A Pubmed search was carried out in October 2022, the term vagina- had to be present in the title, combined with one of the following options: dehiscence, evisceration, rupture, separation, breakdown or herniation. The terms “cesarean”, “labor”, “membrane” and “delivery” were exclusion criteria in the title/abstract to avoid articles concerning obstetric subjects. The following filters were applied: English (for the text language), humans (to exclude animal-conducted studies); abstracts and full texts had to be available. This resulted in 219 results. After going through these articles, we identified: 97 case-reports, 37 retrospective studies, 7 literature reviews, 4 randomized controlled trials, 1 systematic review and 1 combined meta-analysis and systematic review. Three retrospective studies, published between March and May 2023, were later added due to their important cohort size of about 4000 to 6500 patients.

## 4. Review of the Literature

### 4.1. Case Reports

Ninety-seven articles, describing a total of 116 cases concerning vaginal dehiscence (not related to obstetric trauma) were identified and reviewed. The cases were reported between 1981 and 2022 (1981–1990: 2 cases; 1991–2000: 11 cases; 2001–2010: 23 cases; 2011–2022: 80 cases). No gynecological surgical history predisposing to this condition, was found in 13 cases. In the vast majority (103 cases, 88.8%), patients had a surgical history, the most common being total abdominal hysterectomies (TAH) (34 cases, +7 radical abdominal hysterectomies), followed by 22 total laparoscopic hysterectomies (TLH), 15 vaginal hysterectomies (VH), 10 robotically-assisted total laparoscopic hysterectomies (RATLH) (+1 radical RATLH). Other, less frequent previous surgeries were (radical) cystectomies, sacro-colpopexy, resection of a lesion on the vaginal vault, trachelectomy and even one case after a (traumatic) dilatation and curettage. The dehiscence occurred from one week up to thirty years post-operatively. Most commonly (74%), a precipitating factor to the dehiscence was described, sex being the main trigger (51%), followed by defecation (29%), cough (7%), unspecified valsalva (5%), trauma (5%), and weight carry (3%). Evisceration was common (77.8%), but only in 12 cases (10.3%) was an intestinal resection required. Only three cases were managed expectantly, all of the others (except one where reparation was not specified) needed surgical intervention: 49 cases by laparotomy, 44 by vaginal route, 15 by laparoscopy (the first in 1995), 3 combined laparoscopy and vaginal, one case by robotically-assisted laparoscopy. An overview of this information can be found in the supplementary material (Table S1).

This field of research is rapidly evolving, and new clinical cases appear at an increasing pace. Since our literature review, perfomed in October 2022, we have already identified two new case reports (which were not included in our description here above). We anticipate that many new case reports will be published in the coming years.

### 4.2. Retrospective Studies

Forty retrospective studies concerning VCD were identified, published from 2005 to 2023.

The reported rates of VCD varied between 0.02% [4] and 4.2% [5], with other studies falling within this range [3,6,7,8,9,10,11,12,13,14,15,16,17,18,19,20,21,22,23,24,25]. Some studies focused on specific hysterectomy types, providing incidence ranges for each surgical approach, as shown in Table 1.

Different conclusions were drawn when comparing VCD rates among various types of hysterectomy. Some proved a statistically significant higher incidence of VCD in TLH compared to both TAH and VH [6,8,10,16,17,26,27]. Ala Nissilä et al. [3] noticed a rise of cases of VCD, parallel to a rise in the number of total laparoscopic hysterectomies in their institution. Fuchs Weizman et al. [16] also found a higher rate of VCD in RATLH compared to TAH and VH.

Comparisons between TLH and LAVH consistently showed higher VCD rates in TLH [10,15,22]. Notably, Fanning et al. [22] acknowledged significant differences in patient characteristics between TLH and LAVH groups, including age, BMI, comorbidities, blood loss and operative time. These differences were used by the authors to emphasize the fact that a worse outcome should have been expected in the LAVH group, though some of these differences (such as higher age and BMI) could actually be protective. In a subanalysis by Kim et al. [15], TLH had a higher incidence of VCD compared to LAVH. Interestingly, within the TLH group, laparoscopic suture with intracorporeal knotting was found to be more protective against VCD compared to vaginal continuous locking sutures. Uccella et al. [27] also examined different suture methods within the TLH group and found a statistically higher VCD rate in the laparoscopic closure subgroup. When comparing TLH with vaginal closure to TAH or VH, no significant difference in VCD was observed.

Iaco et al. [28] did not find a higher VCD incidence rate according to the route of surgery (TAH, VH and TLH), but TLH only represented 3.5% of the cases. They also compared vaginal cuff left open (where edges were sutured but left open, and the peritoneum closed) vs. closing of the vaginal cuff, and found no statistical difference in dehiscence rates. This was confirmed by Ceccaroni et al. [8].

Some studies comparing TLH with RATLH did not find a statistical difference in VCD incidence [5,9,18,19,23,29]. Most of these only had a small cohort of RATLH. However, Polin et al. [23] recently published a study in which they included 1360 cases of RATLH, and found a slightly higher VCD incidence rate in RATLH (0.66%, compared to 0.32% in TLH and 0.27% in TAH), but which was not significant. More specifically, Das et al. [19] compared laparoscopic and vaginal cuff closure in both TLH and RATLH, and found no superiority of either method.

Unlike in the studies mentioned above, Koo et al. [13] found lower VCD incidence rates for laparoscopic surgery (LAVH and laparoscopic radical hysterectomy) compared to VH and abdominal hysterectomy (TAH and abdominal radical hysterectomy). However, TLH and RATLH were not included in this study because of low TLH and RATLH in the center where the study was done.

The choice of suture type has been studied as a potential influence on VCD risk. Some studies suggested that barbed sutures were associated with lower dehiscence rates compared to braided sutures [18] or braided and monofilament sutures [5]. Barbed sutures were also associated with shorter operative time [31,32], less post-operative bleeding [14], shorter hospital stay [33] and less vaginal cuff granulation at 6 months post-operatively [34]. The use of unidirectional barbed suture for vaginal cuff closure was found to be safe, with no major complications reported (in a cohort of 121 patients) [35]. Fuchs Weizman et al. [16] found a continuous suture to be protective against VCD, but found no difference in comparing braided and barbed thread, nor hand or laparoscopic sewn. On the other hand, Blikkendaal et al. [36] did not find a statistical difference regarding VCD rates when comparing different methods (vaginal or laparoscopic interrupted sutures, laparoscopic running suture) or types (braided and barbed). MacKoul et al. [37] compared the use of absorbable vs non-absorbable sutures, and did not find a statistically significant superiority of either (and the non-absorbable sutures had to be surgically removed after 90 days).

The use of energy has also been explored as a potential factor influencing VCD risk (especially in laparoscopic hysterectomies). Uccella et al. [27] did not find a reduced risk of VCD in TLH when lowering monopolar power, nor when comparing colpotomy methods (cold knife vs monopolar) in TAH. Studies comparing different energy methods, such as Harmonic shears, Ligasure, mono and bipolar energy, and cold knife, could not prove a difference [16,18]. In their study, where significantly more VCD were found in TLH (compared to LAVH), Fanning et al. [22] concluded that since both surgical methods were performed entirely by electrosurgery (including colpotomy), electrosurgery did probably not play a major role in VCD. However, it is important to note that both groups did not use the same energy (harmonic shears in the TLH group, monopolar pencil in the LAVH group), and as mentioned above, both group characteristics differed.

The indication of the surgery might influence the VCD rate. Ceccaroni et al. [8] found malignancy to be a risk factor when compared to prolapse as an indication, while others [13,27] did not find this increased risk for malignancy or prolapse as surgery indications. A recent study on a large cohort of more than 5000 patients also found an increased risk of VCD with malignancy as the indication [24]. Another study (on a cohort of about 6500 patients) showed a higher VCD rate in the minimally invasive surgery group for benign indications, while malignancy was linked to a higher risk of VCD in the TAH group [25]. Radical hysterectomies [29] and the use of adjuvant chemo- or brachytherapy [12] have also been identified as factors for VCD.

Certain factors that generally affect wound healing, such as smoking, diabetes, age, BMI and comobidities, may logically impact VCD rates. Some identified risk factors include low BMI (high BMI is protective), younger age (older age is protective), earlier resumption of sexual activity (which may be linked to the previous risk factor), smoking, longer operative times, and heavier uterine weight [12,19,20,29,38]. However, a recent study on a large cohort of 5072 patients found a high BMI to increase the risk of VCD [24]. Fuchs Weizman et al. [16] found more major complications among women with VCD, including hematoma/hemoperitoneum and wound infection. Klauschie et al. [39], in a study analyzing the histological characteristics of vaginal cuffs from patients with VCD, found evidence of impaired wound healing, including the presence of inflammatory cells and absence of expected post-operative changes in collagen and muscle cells.

Sexual intercourse has been identified as the main trigger event in several studies [3,7,9,21,23,26,37], particularly in younger patients, while VCD may be a spontaneous event in older patients [8]. The time interval to VCD has been noted to be shorter after TLH compared to other surgical routes (TAH, VH) [26,27]. VCD occurrence was also accelerated when vaginal suture was performed compared to laparoscopic suture [15], and in RATLH compared to TLH [29].

Lastly, the surgeon’s experience is expected to influence VCD risk, which was confirmed by Radosa et al. [20], whose study found that less experienced surgeons had a higher risk to VCD. Dauterive et Morris [9] observed a decrease in VCD cases after the surgeon’s first 25 RATLH procedures. Others did not find a significant influence of surgeon volume [12,19]. Chapman et al. [40] organized a simulation for recognition and management of VCD, and found gynecology residents’ knowledge and confidence in recognizing VCD, identifying the need for surgical management, and performing a reduction of prolapsed bowel and vaginal cuff repair to be improved after the training.

### 4.3. Clinical Trials

The first randomized controlled trial (RCT) was published in 2010 by Jeung et al. [41], where they randomized 248 patients with indication to TLH for benign disease into two groups: group 1 where the vaginal cuff was closed by interrupted figure-of-eight sutures, and group 2 where the cuff was closed by a two-layer running suture, using the same thread (Polysorb^®^ No. 1). The main outcome was cumulative incidence to vaginal cuff dehiscence and other major complications, the second goal was identifying possible risk factors for this condition. The two groups did not differ in basic characteristics, nor in operation time and estimated blood loss. VCD was found in two cases in group one and one case in group two, all triggered by sexual intercourse. This was not enough to prove statistical difference. No difference was found in complication rates (vaginal spotting, vaginal bleeding, incisional hernia and vaginal dehiscence) between both groups. Risk factors that were identified to have a significant impact on complications were diabetes, moderate to severe pelvic adhesions, smoking status and low-and-high BMI groups (compared to mid range BMI). Menopausal status did not show an influence.

Landeen et al. [42] published an RCT in 2016 comparing robot vaginal cuff closures in 263 patients. Group 1 underwent a single layer continuous closure (0-Maxon^®^ thread), while group 2 had three additional figure-of-X sutures (Polysorb^®^ 1 thread) placed in addition to the standard protocol. VCD occurred in four patients in total (incidence 1.49%), three in the first group (2.08%) and one in the second group (0.804%), which was statistically significant. No difference was found in secondary outcomes.

The largest RCT was published by Uccella et al. [43] in 2018, comparing transvaginal and laparoscopic closure of the vaginal vault after TLH in 1395 patients. Patients with transvaginal closure had a significantly higher incidence of VCD (2.7% vs. 1%) and of any cuff complication (9.8% vs. 4.7%) compared to laparoscopic closure. Complications that were significantly different included cuff hematoma, post-operative bleeding and infection, the need for reintervention and vaginal cuff resuturing. The trial was recommended to terminate early because of these findings in the interim analysis. Premenopausal status and smoking were independently associated with higher risk of VCD.

The last found RCT, published by Taskin et al. [44] in 2019, was the only one to compare monopolar cut vs coagulation mode in colpotomy in TLH in 199 patients. VCD occurred in only one patient, not allowing us to show statistically significant differences in VCD rates, nor in other vaginal-cuff-related complications.

### 4.4. Systematic Review

The first systematic review was published by Uccella et al. [45] in 2011, in which they included 57 studies for a total of 13,030 patients. They also added their own experience by doing a retrospective analysis on prospective collected data on 665 patients (with both benign and malignant surgery indication). It showed a significantly higher rate of VCD in laparoscopic hysterectomy with laparoscopic and robotic cuff closure, compared to vaginal cuff closure. Though we have to keep in mind that this systematic review was published before other important work from the same authors (being an RCT and meta-analysis and systematic review).

### 4.5. Systematic Review and Meta-Analysis

The only combined meta-analysis and systematic review was published by Uccella et al. [46] in 2021, by the same author who had already published a systematic review, retrospective study and randomized controlled trial about VCD. They included 20 studies, for a total of 19392 patients. They found an overall VCD incidence of 0.53%, but this went up to 1.78% when only the RCT was considered. Sexual intercourse was reported as the trigger event in 64%, and spontaneous evisceration in 12%. Bowel evisceration occurred in 29% of the VCD cases (in the studies that reported it), and the mean time from surgery to evisceration was 68.5 days (range 5–512 days). No definitive conclusions could be drawn on the role of electrosurgery, since no studies satisfied the inclusion criteria. No difference was found in the incidence of VCD between single-layer suturing and reinforced or double-layer suturing in TLH and RATLH. Regarding the role of training in endoscopic suturing, only one study was found that compared it [12], but it was not included in the meta-analysis because it concerned hysterectomies for malignancy. Barbed sutures were studied in 11 studies, and found a VCD rate that was significantly lower in barbed vs polyglactin or other types of sutures (0.4% vs. 2%, 95% CI 0.11–0.5, respectively), though this difference was not statistically significant anymore when only TLH were compared (excluding RATLH). When comparing vaginal cuff closure and laparoscopic cuff closure after TLH, the latter was associated with a lower risk of VCD, as was found in the large RCT from the same authors, discussed above [43].

## 5. Discussion

When Ramirez and Klemer published the first review concerning vaginal cuff evisceration in 2002 [47], they identified 59 cases of vaginal evisceration occurring after hysterectomies, and no other studies were discussed. Twenty years later, we collected 116 cases of vaginal cuff dehiscence, but fortunately also had the possibility to review retrospective studies, a few randomized controlled trials, a systematic review and even one meta-analysis and systematic review concerning this rare subject. However, despite having more information than before, the lack of high-quality evidence leaves many questions unanswered. Conducting randomized clinical trials is challenging due to the low incidence rate and difficulty in collecting sufficient cases to have sufficient power, and a general underreporting of the cases is still very likely.

### 5.1. Vaginal Cuff Closure Recommendation

The only level-one recommendation that could be done by the only systematic review and meta-analysis [46] is the laparoscopic approach for the vaginal cuff closure, which lowered the VCD incidence. This might be due to the inclusion of the peritoneum of the pouch of Douglas (which might reduce post-operative oozing of blood from the vagina, reducing risk of hematoma formation and inflammation at that level), better targeting of vaginal tissue (due to the improved visualization), and might sometimes offer an easier access (for example in obese patients or in long and narrow vaginas).

### 5.2. Laparoscopy Risk Factors

The use of laparoscopy and robot-assisted laparoscopy may contribute to the rising levels of vaginal cuff dehiscence [3,16]. When reporting three cases of VCD after laparoscopic hysterectomy, Nezhat et al. [48] suspected that this could be linked to either the use of electrosurgery (they therefore recommended the placement of sutures in viable tissue and to minimize the use of electrosurgery to avoid over-desiccating the tissue) or to the quicker recovery associated with a laparoscopic approach, which would allow a swift return to everyday activities, including resuming sexual intercourse.

### 5.3. Robotic Surgery Risk Factors

Despite the fact that we did not find a study that found statistically higher rates of VCD between TLH and RATLH, a 2011 systematic review [45] found a robotic approach to hysterectomy to be associated with an increase in VCD, even when compared to TLH. One retrospective study [49] compared closure of the vaginal cuff with monofilament vs barbed suture within RATLH, found no difference in vaginal spotting or bleeding, and no cases of VCD or cuff cellulitis were reported (yet the study only had a total of 134 patients). Muffly et al. [50] conducted a study where the knot integrity between conventional (by hand) and robotic (da Vinci robot) knotting was compared with three different sutures (polyester coated with polybutyrate = Ethibond^®^, polyglactin 910-dyed suture = Vicryl^®^, and polypropylene = Prolene^®^). They found robotically-tied polyglactin 910 to be significantly weaker than all other robotically and conventionally tied knots, as well as tying modality and material interaction to be significant too, suggesting that the effect of suture material varied depending on the tying modality

### 5.4. Suture Type Recommendation

Both our review of retrospective studies, and the meta-analysis and systematic review of Uccella et al. [46], found barbed sutures to lower the risk of VCD (although not significantly when only compared in TLH). This better outcome could result from the better tensile strength held by barbed sutures compared to polyglactin sutures [14,36]. The latter study also found a longer time to dehiscence in barbed sutures, compared to polyglactin sutures (73 days vs. 29 days, respectively). Though a study [35] assured the safety of the use of barbed sutures, two case-reports of small bowel volvulus and obstruction resulting from barbed sutures were published [51,52]. Animal studies showed conflicting results, with one study in ewes showing no difference in adhesion formation between barbed and polyglactin sutures [53], while a similar experiment in rats found more adhesion formation and increased presence of inflammatory cells with barbed sutures [54].

### 5.5. Electrosurgical Risk Factors

Even though the discussed meta-analysis and systematic review did not find studies that met the inclusion criteria, nor did the discussed retrospective studies prove an influence of electrosurgery on VCD rates, it remains a widely discussed possible risk factor. An RCT comparing “Valleylab mode” (V mode) to cut/coagulation electrothermal energy found no difference in terms of median depth of thermal injury, nor in the development of granulation tissue, induration, infection or dehiscence at the vaginal cuff at 4 weeks, 3 months and 6 months post-operatively [55]. Another retrospective study, comparing cutting mode (at 30 W) and coagulation mode (at 40 W) for colpotomy in RATLH, found significantly more complications in the coagulation mode [56]. A histopathological assessment of laparoscopic colpotomy in swine found bipolar energy to cause the greatest tissue damage, which was significantly more than ultrasonic, which had the least tissue damage [57]. Monopolar was in between both, but did not have significantly more damage than ultrasonic.

### 5.6. General Recommendations for Diagnosis and Management

Studies on the subject have become more frequent over the years, but because of the low incidence, high quality evidence remains scarce and further research is needed to have a better knowledge of the individual impact of the possible risk factors. This review is limited by the fact of it being a narrative review, thus not permitting us to give precise recommendations. However, it presents an overview of the studies concerning VCD, as well as highlights some of the possible risk and protective factors.

Good quality evidence shows a protective influence of a laparoscopic approach for the vaginal cuff closure concerning VCD and other cuff-related complications. Barbed sutures might be protective, though possible complications on these sutures should be kept in mind. Extensive electrocauterization on the vaginal cuff should be avoided, and the sutures should be placed in healthy, viable tissue. Since sexual intercourse is often identified as being a trigger, patients who underwent a hysterectomy (especially if they are known to have additional risk factors), should wait to engage in intercourse until 4 to 6 weeks minimum post-operatively.

Patients presenting at their hysterectomy post-operative consultation should be examined (by digital or speculum examination) to confirm the correct healing of the vault. Even if this examination is normal, a patient presenting with lower abdominal pain, blood or abnormal vaginal discharge, or a vaginal pressure, should be examined to rule out VCD and evisceration. Medical imaging and complete bloodwork can be considered, although the diagnosis will mainly be made upon physical examination.

Eoh et al. [25] designed a flowchart for the management of patients suspected of having VCD. For small dehiscence of less than 1cm, a conservative management may be considered, but the need of antibiotics should be evaluated, and presence of cuff hematoma and abscess should be ruled out. However, in case of total vault dehiscence, evisceration or severe bleeding and unstable vital signs, an urgent surgical intervention is needed. It is then also recommended to start a treatment with a broad-spectrum antibiotic. Various approaches (abdominal, vaginal, laparoscopic, or combined) have been successful in managing VCD. In 1996, Nezhat et al. were the first ones (found in our literature review) to propose a laparoscopic approach to VCD repair [48]. Narducci et al. [58] suggested a combined laparoscopic and vaginal approach with an omental flap for the VCD repair in two of their oncologic cases, aiming to improve tissue vascularization compromised by radical surgery or radiotherapy. The choice should be made by the treating clinician in an approach in which he feels confident, though if bowel necrosis or perforation is suspected, an approach allowing careful examination of the bowel should be preferred.

In the absence of bowel necrosis and with timely management, VCD has a good prognosis. Recurrences have been described in a minority of cases. Prompt recognition and adequate management of VCD is crucial to avoid further complications that can raise the morbidity related to this complication.

## 6. Conclusions

Vaginal cuff dehiscence, especially when associated with evisceration, remains a rare, but potentially life-threatening condition. Even when treated promptly and successfully, patients are often very emotionally impacted by this diagnosis. Clinicians should keep the possible influential factors, including patient-specific risk factors, and this diagnosis in mind when treating patients by hysterectomy or other possible predisposing surgical interventions.

## Figures and Tables

**Table 1:** Vaginal cuff dehiscence incidence by surgical approach in retrospective studies.

Surgical Approach	Lowest VCD Incidence Range	Highest VCD Incidence Range	References within Range
Total laparosopic hysterectomy (TLH)	0.2%	5.42%	[3,6,9,10,11,13,14,15,22,23,24,26,27,28]
Vaginal hysterectomy (VH)	0.05%	0.14%	[3,6,10,13,26,28]
Laparoscopically-assisted vaginal hysterectomy (LAVH)	0.11%	1.68%	[3,10,15]
Total abdominal hysterectomy (TAH)	0.02%	1%	[3,6,10,13,23,24,25,26,28]
Robotically-assisted total laparoscopic hysterectomy (RATLH)	0.4%	4.1%	[9,12,21,23,24,27,29,30]

## Data Availability

Not applicable.

## Supplementary Materials

The following supporting information can be downloaded at: https://www.mdpi.com/article/10.3390/jcm12134187/s1, Table S1: Table of case reports of vaginal cuff dehiscence and list.
