# Supplementary material for: Vaginal Cuff Dehiscence: Two Case Reports and a Review of the Literature
Source: J Clin Med. 2023 Jun 21;12(13):4187. doi: 10.3390/jcm12134187 (PMC10342889; doi:10.3390/jcm12134187)
Supplement: Supplementary file 1 [file jcm-12-04187-s001.zip › jcm-2376784-supplementary.pdf]

Table S1. Table of case reports of vaginal cuff dehiscence and list.

Article number	Name 1st author	Year publication	Surgery history	Type surgery	Weeks since surgery	Trigger	Reparation type	Evisceration	Intestine resection	Age patient	Menopausal status	Prolaps	Oncology	Type oncology treatment	Recurrence	Supplementary information
1	A. Ernest	2014	No	NA	NA	Sex	Laparotomy	Yes	No	28	No	No	No	NA	No	
2	Agrawal	2020	Yes	TAH	7	Sex	Laparoscopy	Yes	No	45	No	No	No	NA	No	
3	Akdemir	2014	Yes	RATLH	27	Sex	Vaginal	Yes	No	55	Yes	No	Yes	NA	No	
4	Alfraidi	2022	Yes	LH	40	Defaecation	Laparotomy	Yes	No	54	Yes	No	No	NA	No	
5	Apicella	2022	Yes	RATLH	11	Sex	Vaginal	Yes	No	49	NS	No	No	NA	No	
6	Arabadzhieva	2022	No	NA	NA	Spontaneous	Laparotomy	Yes	No	80	Yes	Yes	No	NA	No	Pre-existing prolaps
7	Austin	2013	No	NA	NA	Sex	LPS + vag	No	No	23	No	No		NA	No	
8	Baines	2017	Yes	TAH	884	Defaecation	LPS + vag	Yes	No	55	Yes	No	No	NA	No	
9	Ben Safta	2017	Yes	TAH	572	Cough	Laparotomy	Yes	No	68	Yes	No	Yes	CT + RT	No	
10	Bleull	2017	Yes	TAH	10	Sex	Vaginal	Yes	No	39	No	No	No	NA	No	
11	Boersen	2019	Yes	LH	11	Sex	No reparation	No	No	36	No	No	No	NA	No	Use GnRH agonist
11	Boersen	2019	Yes	LH	9	Sex	Vaginal	No	No	39	No	No	No	NA	No	Use GnRH agonist
11	Boersen	2019	Yes	LH	31	Sex	No reparation	No	No	35	No	No	No	NA	No	Use GnRH agonist
11	Boersen	2019	Yes	LH	9	Sex	No reparation	No	No	46	No	No	No	NA	No	
12	Bozkurt	2008	No	NA	NA	Valsava other	Vaginal	Yes	No	73	Yes	Yes	No	NA	No	
13	Burkett	2007	Yes	TAH	3	Spontaneous	Vaginal	No	No	63	Yes	No	Yes	HIPEC	No	
14	Canturk	2017	Yes	RATLH	1	Defaecation	Robot	Yes	No	63	Yes	No	Yes	NA	No	
15	Cerqui	1998	Yes	VH	36	Valsava other	Laparotomy	Yes	No	61	Yes	Yes	No	NA	No	
16	Chan	2021	Yes	TAH	780	NS	Laparotomy	Yes	No	97	Yes	Yes	No	NA	No	
17	Chen	2020	Yes	RATLH	22	Sex	Vaginal	No	No	69	Yes	No	Yes	NA	No	
18	Chhabra	2013	Yes	Exenteration + TAH	13	NS	Laparotomy	Yes	No	51	Yes	No	Yes	NA	No	
19	Chong	2010	Yes	LH	31	Defaecation	Laparoscopy	Yes	No	50	Yes	No	Yes	CT	No	
20	Codd	2010	Yes	VH	156	Defaecation	Laparotomy	Yes	No	63	Yes	Yes	No	NA	No	Vaginal vault biopsy 3 days earlier
21	Cruickshank	2018	Yes	VH	1040	Spontaneous	Laparotomy	Yes	No	92	Yes	Yes	No	NA	No	
22	Dawlatly	1999	Yes	TAH	1	Defaecation	Laparotomy	Yes	No	45	No	No	Yes	NA	No	Vaginal apex left open
23	De Boisredon	2021	Yes	VH	364	Spontaneous	Vaginal	Yes	No	80	Yes	Yes	No	NA	No	
24	Dharmalingham	2004	Yes	TAH	31	Cough	Laparotomy	Yes	Yes	58	Yes	No	Yes	NA	No	
25	Eid-Arimoku	2011	Yes	LH	40	Sex	Laparotomy	No	No	35	No	No	No	NA	No	Peritonitis
26	Escobar	2016	Yes	RATLH	66	Sex	Vaginal	Yes	No	43	Yes	No	Yes	NA	No	
26	Escobar	2016	Yes	RATLH	49	NS	Vaginal	No	No	51	Yes	No	Yes	CT	No	
26	Escobar	2016	Yes	RATLH	49	NS	Vaginal	No	No	70	Yes	No	Yes	BT + RT	No	
27	Feiner	2003	Yes	VH	780	Defaecation	Vaginal	Yes	No	83	Yes	Yes	No	NA	No	
28	Ferrera	1999	Yes	TAH	13	NS	Laparotomy	Yes	No	38	No	No	No	NA	No	
28	Ferrera	1999	Yes	TAH	31	NS	Laparotomy	Yes	Yes	51	Yes	Yes	No	NA	No	
29	Frank	2021	Yes	Vaginal vault surgery (resection)	6	Sex	Vaginal	Yes	No	37	No	No	No	NA	Yes	LH 5 years earlier
29	Frank	2021	Yes	Vaginal vault surgery (resection)	10	Sex	Laparoscopy	No	No	44	No	No	No	NA	Yes	LH + rectum resection 16 months earlier
30	Gheewala	2015	No	NA	NA	Valsava other	Laparotomy	Yes	No	60	Yes	Yes	No	NA	No	
31	Ginsberg	1998	Yes	VH	NS	NS	Laparotomy	Yes	Yes	62	Yes	Yes	No	NA	No	
32	Gold	2021	Yes	VH	52	Cough	Laparotomy	Yes	No	72	Yes	Yes	No	NA	No	
33	Gujar	2011	Yes	TAH	104	Sex	Laparotomy	No	No	40	No	No	No	NA	No	
34	Guttmann	1990	Yes	TAH	1560	Defaecation	Laparotomy	Yes	Yes	83	Yes	No	No	NA	No	Subtotal HRT 30 years earlier, trachelectomy 20 years earlier
35	Jimi	2017	Yes	LH	27	Sex	Laparoscopy	Yes	No	51	Yes	No	No	NA	No	
36	Kahramanoglu	2016	Yes	LH	6	Sex	Vaginal	Yes	No	32	No	No	Yes	NA	No	
37	Kalogeropoulos	2018	Yes	Trachelectomy	4	Sex	Laparotomy	No	No	45	No	No	No	NA	No	LH 2 years earlier
38	Kambouris	1981	Yes	VH + colporaphia anterior	13	Trauma	Laparotomy	Yes	Yes	70	Yes	Yes	No	NA	No	VH 2 years earlier
39	Kang	2009	Yes	Radical TAH	364	Defaecation	Vaginal	Yes	No	40	Yes	No	Yes	BT + RT	No	
40	Kim	2002	Yes	Radical TAH	7	Defaecation	Vaginal	Yes	No	57	Yes	No	Yes	NA	No	
41	Kim	2019	Yes	D&C	13	Trauma	Laparotomy	Yes	No	26	No	No	No	NA	No	10 days postoperatively had to have subtotal HRT (as repair of damage D&C)
42	Kiziloglu	2017	Yes	TAH	312	NS	Laparotomy	Yes	No	80	Yes	No	No	NA	No	
43	Kumar	2017	Yes	TAH	104	NS	Laparotomy	No	No	60	Yes	No	Yes	CT + RT	No	
44	Lan	2017	Yes	TAH	31	Sex	Vaginal	Yes	No	41	Yes	No	Yes	CT	No	
45	Lawson	2011	Yes	LH	260	Cough	Laparotomy	Yes	Yes	63	No	Yes	No	NA	No	
46	Lee	2022	Yes	Radical TAH	11	Defaecation	Vaginal	Yes	No	65	Yes	No	Yes	NA	No	
47	Lin	2019	Yes	Radical cystectomy	16	NS	Vaginal	No	No	55	Yes	Yes	Yes	NA	No	Bladdercarcinoma
47	Lin	2019	Yes	Radical cystectomy	27	NS	Vaginal	No	No	63	Yes	Yes	Yes	NA	Yes	Bladdercarcinoma, partial vaginectomy for recurrence
47	Lin	2019	Yes	Radical cystectomy	19	Cough	Vaginal	Yes	No	73	Yes	Yes	Yes	NA	No	Bladdercarcinoma
47	Lin	2019	Yes	Radical cystectomy	151	NS	Vaginal	Yes	No	73	Yes	Yes	No	NA	No	Bladdercarcinoma
47	Lin	2019	Yes	Radical cystectomy	11	NS	Vaginal	No	No	79	Yes	Yes	Yes	Neoadjuvant CT	No	Bladdercarcinoma
48	LLedo	2002	Yes	VH	156	Weight carry	Laparoscopy	Yes	No	58	Yes	Yes	No	NA	No	Enterocoele repair 2 years earlier
49	Manchanda	2005	No	NA	16	Sex	Vaginal	No	No	16	No	No	No	NA	No	
50	Mastrolia	2014	Yes	VH	520	NS	Vaginal	Yes	No	58	Yes	Yes	No	NA	No	Vaginoplasty 3 years post HRT, sacrocolpofixation 5 years post HRT
51	Matsuhashi	2017	Yes	Radical TAH	5	NS	Laparoscopy	Yes	No	62	Yes	No	Yes	NA	No	Endometrial carcinoma
52	McCullough	2012	No	NA	NA	Sex	Laparoscopy	No	No	17	No	No	No	NA	No	
53	McMasters	2019	Yes	LH	8	Valsava other	Vaginal	Yes	No	45	No	No	No	NA	No	
54	Minguez	2011	Yes	SCP	40	NS	Laparotomy	Yes	No	53	Yes	Yes	No	NA	No	VH 4 years earlier + other prolaps surgery
55	Missori	2022	Yes	VH	624	Defaecation	Laparotomy	Yes	Yes	72	Yes	No	No	NA	No	
56	Mohamed Salad	2022	Yes	TAH	416	Defaecation	Laparotomy	Yes	No	55	Yes	No	No	NA	No	
57	Muraoka	2012	Yes	VH	208	Weight carry	Laparotomy	Yes	No	81	Yes	Yes	No	NA	No	
58	Narducci	2003	Yes	Radical TAH	18	Sex	Vaginal	Yes	No	34	Yes	No	Yes	BT	Yes	Cervixcarcinoma, second surgery laparoscopic repair with omental flap
58	Narducci	2003	Yes	Radical TAH	9	Trauma	Laparoscopy	Yes	No	74	Yes	No	Yes	BT	No	Endometrial carcinoma, tear on BT
59	Nasr	2005	Yes	TAH	988	Weight carry	Laparotomy	Yes	No	75	Yes	No	No	NA	No	
60	Negrete	2021	Yes	SCP	9	Cough	Laparotomy	Yes	Yes	50	No	Yes	No	NA	No	
61	Newell	2021	Yes	LH	18	Sex	Laparoscopy	Yes	No	40	No	No	No	NA	No	
62	Nezhat	1996	Yes	LH	18	Defaecation	Vaginal	Yes	No	42	No	No	No	NA	No	
62	Nezhat	1996	Yes	LH	18	Sex	Laparoscopy	Yes	No	43	No	No	No	NA	No	
62	Nezhat	1996	Yes	LH	22	Sex	Vaginal	Yes	No	40	No	No	No	NA	No	
63	Nguyen	2013	Yes	TAH	162	NS	Laparotomy	No	No	50	No	No	No	NA	No	Presence ovarian cyst 10cm
64	Nguyen	2013	Yes	RATLH	18	Sex	Laparotomy	Yes	No	45	No	No	No	NA	No	
64	Nguyen	2013	Yes	RATLH	9	Sex	Laparotomy	Yes	No	44	No	No	No	NA	No	
65	Nikolopoulos	2013	Yes	TAH	49	Defaecation	Vaginal	Yes	No	56	Yes	No	No	NA	No	
66	Orito	2012	Yes	TAH	156	Sex	Laparotomy	Yes	No	53	Yes	No	Yes	NA	No	Endometrial carcinoma
67	Parra	2010	Yes	TAH	1560	Defaecation	Laparotomy	Yes	No	75	Yes	Yes	No	NA	No	2x repair of cystocoele
68	Partsinevelos	2009	Yes	VH	208	Defaecation	Laparotomy	Yes	No	70	Yes	Yes	No	NA	No	
69	Pereira	2012	Yes	TAH	1560	Defaecation	Vaginal	Yes	No	66	Yes	Yes	No	NA	No	
70	Pinto	2022	No	NA	NA	Defaecation	Laparotomy	Yes	No	63	Yes	Yes	No	NA	No	History of vaginal laceration at delivery
71	Rajesh	2008	Yes	TAH	260	Sex	Laparotomy	Yes	No	55	Yes	No	No	NA	No	
72	Rana	2019	Yes	LH	52	Spontaneous	Laparotomy	Yes	No	91	Yes	No	Yes	NA	No	
73	Ribeiro	2016	Yes	TAH	52	Spontaneous	Laparotomy	Yes	No	76	Yes	No	No	NA	Yes	Omental evisceration 2 years later
74	Robinson	2009	Yes	RATLH	8	Sex	Vaginal	Yes	No	48	Yes	No	No	NA	Yes	Recurrence treated vaginally 7 weeks later
74	Robinson	2009	Yes	Radical RATLH	7	Sex	Vaginal	Yes	No	57	Yes	No	Yes	NA	No	Cervixcarcinoma
75	Rogers	2019	Yes	TAH	NS	Defaecation	Laparotomy	Yes	No	83	Yes	Yes	No	NA	No	
76	Schefter	2018	No	NA	NA	Defaecation	Laparotomy	Yes	Yes	89	Yes	Yes	No	NA	No	Colostomy + SCP
77	Schreiner	2017	No	NA	NA	Defaecation	Vaginal	Yes	No	40	No	Yes	No	NA	No	SLE treated by glucocorticoids for 5 years
78	Sendy	2020	Yes	LH	13	Sex	Laparoscopy	Yes	No	40	No	No	No	NA	No	
79	Shehata	2019	Yes	TAH	1560	NS	Vaginal	No	No	66	Yes	Yes	Yes	NA	No	Coloncarcinoma
80	Siddiqui	2011	No	NA	NA	Trauma	Laparotomy	Yes	No	79	Yes	Yes	No	NA	No	Pessary use
81	Sinclair	2010	Yes	VH	208	NS	LPS + vag	Yes	No	75	Yes	Yes	No	NA	No	
82	Sokol	2007	Yes	Radical TAH	156	NS	Vaginal	No	No	36	No	Yes	Yes	CT	No	Cervixcarcinoma, incarcerated omentum
83	Solberg	2022	Yes	VH	104	Defaecation	NS	Yes	No	31	No	No	No	NA	No	
84	Somkuti	1994	Yes	TAH	13	Sex	Vaginal	Yes	No	31	No	No	No	NA	No	
84	Somkuti	1994	Yes	TAH	18	Sex	Vaginal	Yes	No	36	No	No	Yes	NA	No	Cervixcarcinoma in situ
84	Somkuti	1994	Yes	TAH	10	Sex	Vaginal	Yes	No	30	Yes	No	No	NA	No	
85	Stabile	2021	No	NA	NA	Sex	Laparoscopy	Yes	No	21	No	No	No	NA	No	
86	Sterck	2019	Yes	LH	13	Sex	Vaginal	No	No	40	No	No	No	NA	No	
87	Szymczak	2020	Yes	Laparoscopic pectopexy	63	Spontaneous	Laparotomy	Yes	No	63	Yes	Yes	No	NA	No	VH 8 years earlier
88	Thomopoulos	2016	Yes	LH	13	Sex	Laparoscopy	Yes	No	49	Yes	No	No	NA	No	
89	Toh	2019	Yes	Radical TAH + cystectomy	NS	NS	Vaginal	Yes	No	66	Yes	Yes	Yes	NA	No	Bladdercarcinoma
90	Vardar	2021	Yes	LH	9	Sex	Vaginal	No	No	40	No	No	No	NA	No	
90	Vardar	2021	Yes	LH	11	Sex	Laparoscopy	No	No	40	No	No	No	NA	No	
91	Walsh	2007	Yes	LH	18	Sex	Laparotomy	Yes	No	47	Yes	No	No	NA	No	
92	Wan Hassan	2021	Yes	Radical cystectomy + TAH	104	Spontaneous	Vaginal	Yes	No	78	Yes	No	Yes	NA	No	Bladdercarcinoma
93	Woo	2012	Yes	TAH + SCP	312	Defaecation	Laparotomy	Yes	Yes	72	Yes	Yes	No	NA	No	Transvaginal mesh repair 3 years before presentation
94	Yaakovian	2008	Yes	TAH	27	Sex	Laparoscopy	Yes	No	42	No	No	No	NA	No	
95	Yanar	2019	Yes	TAH	156	Spontaneous	Laparotomy	Yes	Yes	89	Yes	Yes	No	NA	No	
96	Yuce	2005	Yes	TAH	8	Sex	Vaginal	Yes	No	42	No	No	No	NA	No	
97	Zhou	2018	No	NA	NA	Defaecation	Laparotomy	Yes	Yes	74	Yes	Yes	No	NA	No	

[1] E. A, E. M, and G. K, “Post-coital posterior fornix perforation with vaginal evisceration,” *BMC Womens Health*, vol. 14, Nov. 2014, doi: 10.1186/s12905-014-0141-6.
[2] A. Agrawal, K.-G. Huang, and M. C. V. Mendoza, “Emergency laparoscopic repair of coitus-induced vaginal cuff dehiscence: a case report,” *J. Med. Case Reports*, vol. 14, no. 1, p. 40, Mar. 2020, doi: 10.1186/s13256-020-02362-4.
[3] A. Akdemir, E. Taylan, A. M. Ergenoğlu, A. Ö. Yeniel, F. Şendağ, and M. K. Öztekin, “Vaginal cuff dehiscence with bowel evisceration after robotic hysterectomy,” *J. Turk. Soc. Obstet. Gynecol.*, vol. 11, no. 4, pp. 249–251, Dec. 2014, doi: 10.4274/tjod.47640.
[4] R. Alfraidi, N. Abdulaaly, A. Alharbi, H. Almodhaiberi, B. Ali, and H. Sabagh, “Transvaginal small bowel evisceration: Case report and review of literature,” *Int. J. Surg. Case Rep.*, vol. 96, p. 107322, Jun. 2022, doi: 10.1016/j.ijscr.2022.107322.
[5] M. Apicella, M. Mayrink, C. D. Rajadhyaksha, and D. A. Farcy, “A Case of Transvaginal Small Bowel Evisceration following Hysterectomy with Discussion of Emergency Department Diagnosis and Management,” *Case Rep. Emerg. Med.*, vol. 2022, p. 1334302, 2022, doi: 10.1155/2022/1334302.
[6] E. Arabadzhieva, D. Bulanov, Z. Shavalov, A. Yonkov, and S. Bonev, “Spontaneous transvaginal intestinal evisceration in case of long-standing uterine prolapse,” *BMC Surg.*, vol. 22, no. 1, p. 157, May 2022, doi: 10.1186/s12893-022-01615-x.
[7] J. M. Austin, C. M. Cooksey, L. L. Minikel, and E. F. Zaritsky, “Postcoital vaginal rupture in a young woman with no prior pelvic surgery,” *J. Sex. Med.*, vol. 10, no. 8, pp. 2121–2124, Aug. 2013, doi: 10.1111/j.1743-6109.2012.02682.x.
[8] G. Baines, S. R. Jackson, and N. Price, “Laparoscopic management of spontaneous vaginal vault dehiscence and bowel evisceration 17 years following total abdominal hysterectomy,” *Gynecol. Surg.*, vol. 14, no. 1, p. 1, 2017, doi: 10.1186/s10397-017-1004-6.
[9] Y. Ben Safta *et al.*, “Vaginal cuff dehiscence and evisceration 11 years after a radical hysterectomy: A case report,” *Int. J. Surg. Case Rep.*, vol. 41, pp. 234–237, Jan. 2017, doi: 10.1016/j.ijscr.2017.10.045.
[10] S. Bleull, H. Smith, and R. Shapiro, “Transvaginal Management of Vaginal Cuff Dehiscence with Bowel Evisceration following Delayed Diagnosis,” *Case Rep. Obstet. Gynecol.*, vol. 2017, p. 4985382, 2017, doi: 10.1155/2017/4985382.
[11] Z. Boersen, C. I. M. Aalders, E. R. Klinkert, J. W. M. Maas, and A. W. Nap, “Vaginal Cuff Dehiscence After Endometriosis Surgery,” *JSLS*, vol. 23, no. 3, p. e2019.00018, 2019, doi: 10.4293/JSLS.2019.00018.
[12] N. Bozkurt, U. Korucuoglu, Y. Bakirci, U. Yilmaz, O. Sakrak, and H. Guner, “Vaginal evisceration after trauma unrelated to previous pelvic surgery,” *Arch. Gynecol. Obstet.*, vol. 279, no. 4, pp. 595–597, Apr. 2009, doi: 10.1007/s00404-008-0768-3.
[13] A. M. Burkett, D. E. Cohn, and L. J. Copeland, “Vaginal evisceration during intraperitoneal chemotherapy for advanced ovarian cancer,” *Gynecol. Oncol.*, vol. 104, no. 2, pp. 491–493, Feb. 2007, doi: 10.1016/j.ygyno.2006.10.029.
[14] M. Canturk, V. Ozben, M. F. Kose, and B. Baca, “Robotic repair of vaginal evisceration after hysterectomy and the role of intraoperative near-infrared fluorescence imaging,” *J. Robot. Surg.*, vol. 11, no. 3, pp. 383–386, Sep. 2017, doi: 10.1007/s11701-017-0688-y.
[15] A. J. Cerqui, M. Haran, and S. M. Collier, “A case of transvaginal evisceration,” *Aust. N. Z. J. Obstet. Gynaecol.*, vol. 38, no. 2, pp. 229–231, May 1998, doi: 10.1111/j.1479-828x.1998.tb03011.x.
[16] A. K. Y. Chan, O. Oluwajobi, A. Ehsan, and F. Tahmasebi, “Transvaginal Evisceration of the Small Bowel More Than 15 Years After Abdominal Hysterectomy and Vaginal Surgery,” *Cureus*, vol. 13, no. 3, p. e13955, Mar. 2021, doi: 10.7759/cureus.13955.
[17] H.-Y. Chen, B.-C. Sheu, and W.-C. Chang, “Prolapsed epiploica of bowel after robotic hysterectomy: A case report,” *Ann. Med. Surg. 2012*, vol. 60, pp. 146–148, Dec. 2020, doi: 10.1016/j.amsu.2020.10.055.
[18] S. Chhabra and P. Hegde, “Spontaneous transvaginal bowel evisceration,” *Indian J. Urol. IJU J. Urol. Soc. India*, vol. 29, no. 2, pp. 139–141, Apr. 2013, doi: 10.4103/0970-1591.114038.
[19] G. O. Chong, D. G. Hong, Y. L. Cho, I. S. Park, and Y. S. Lee, “Vaginal evisceration after total laparoscopic radical hysterectomy in cervical cancer,” *Am. J. Obstet. Gynecol.*, vol. 202, no. 3, pp. e7–e8, Mar. 2010, doi: 10.1016/j.ajog.2009.12.009.
[20] R. J. Codd, B. Scourfield, S. Chakravarthy, and G. L. Williams, “Small bowel trans-vaginal evisceration following vault biopsy: general surgeons beware!,” *Ann. R. Coll. Surg. Engl.*, vol. 92, no. 7, pp. W6-7, Oct. 2010, doi: 10.1308/147870810X12699662981870.
[21] L. Cruickshank, A. Amin, and A. DeSilva, “Small bowel evisceration: a late complication following vaginal hysterectomy,” *J. Obstet. Gynaecol. J. Inst. Obstet. Gynaecol.*, vol. 38, no. 5, p. 733, Jul. 2018, doi: 10.1080/01443615.2018.1444397.
[22] B. Dawlatly, O. Lavie, and A. Lopes, “Transvaginal evisceration of small bowel after radical hysterectomy and pelvic lymphadenectomy,” *Gynecol. Oncol.*, vol. 73, no. 1, pp. 165–166, Apr. 1999, doi: 10.1006/gyno.1998.5294.
[23] M. De Boisredon, F. Petitperrin, and C. Henry, “Emergency colpocleisis in a vaginal prolapse complicated by bowel evisceration,” *J. Visc. Surg.*, vol. 158, no. 5, pp. 452–454, Oct. 2021, doi: 10.1016/j.jviscsurg.2021.01.002.
[24] M. Dharmalingam, J. O. Greenhalf, and K. M. Smith, “Vaginal evisceration following total abdominal hysterectomy,” *J. Obstet. Gynaecol. J. Inst. Obstet. Gynaecol.*, vol. 24, no. 2, pp. 194–195, Feb. 2004, doi: 10.1080/01443610410001653434.

[25] L. Eid-Arimoku and V. Trompetas, “Postcoital vaginal rupture after hysterectomy presenting as generalised peritonitis,” *Ann. R. Coll. Surg. Engl.*, vol. 93, no. 7, pp. e136-137, Oct. 2011, doi: 10.1308/147870811X602140.
[26] P. A. Escobar, G. M. Gressel, G. L. Goldberg, and D. Y.-S. Kuo, “Delayed Presentation of Vaginal Cuff Dehiscence after Robotic Hysterectomy for Gynecologic Cancer: A Case Series and Review of the Literature,” *Case Rep. Obstet. Gynecol.*, vol. 2016, p. e5296536, Mar. 2016, doi: 10.1155/2016/5296536.
[27] B. Feiner, A. Lissak, R. Kedar, O. Lefel, and O. Lavie, “Vaginal evisceration long after vaginal hysterectomy,” *Obstet. Gynecol.*, vol. 101, no. 5 Pt 2, pp. 1058–1059, May 2003, doi: 10.1016/s0029-7844(02)02336-0.
[28] P. C. Ferrera and L. G. Thibodeau, “Vaginal evisceration,” *J. Emerg. Med.*, vol. 17, no. 4, pp. 665–667, Aug. 1999, doi: 10.1016/s0736-4679(99)00054-2.
[29] G.-P. Frank, J. Rhemrev, M. Westerterp, and J. English, “Recurrent vaginal cuff dehiscence after surgery for endometriosis: a technique for laparoscopic repair with an omental flap,” *BMJ Case Rep.*, vol. 14, no. 3, p. e239540, Mar. 2021, doi: 10.1136/bcr-2020-239540.
[30] U. Gheewala, A. Agrawal, R. Shukla, R. Bhatt, and S. Srivastava, “Transvaginal small bowel evisceration in known case of uterine prolapse due to trauma,” *J. Clin. Diagn. Res. JCDR*, vol. 9, no. 1, pp. PD09-10, Jan. 2015, doi: 10.7860/JCDR/2015/10345.5411.
[31] D. A. Ginsberg, E. S. Rovner, and S. Raz, “Vaginal evisceration,” *Urology*, vol. 51, no. 1, pp. 128–129, Jan. 1998, doi: 10.1016/s0090-4295(97)00477-9.
[32] R. S. Gold, H. Amir, and A. Groutz, “Late Spontaneous Bowel Evisceration through the Vaginal Vault after Vaginal Hysterectomy,” *Isr. Med. Assoc. J. IMAJ*, vol. 23, no. 2, pp. 126–127, Feb. 2021.
[33] N. N. Gujar, R. K. Choudhari, G. R. Choudhari, N. M. Bagali, M. B. Bendre, and S. B. Adgale, “Coitus induced vaginal evisceration in a premenopausal woman: a case report,” *Patient Saf. Surg.*, vol. 5, no. 1, p. 6, Apr. 2011, doi: 10.1186/1754-9493-5-6.
[34] A. Guttman and M. Afilalo, “Vaginal evisceration,” *Am. J. Emerg. Med.*, vol. 8, no. 2, pp. 127–128, Mar. 1990, doi: 10.1016/0735-6757(90)90199-a.
[35] T. Jimi *et al.*, “A case of laparoscopy-assisted vaginal cuff suturing for vaginal cuff dehiscence after total laparoscopic hysterectomy,” *Int. J. Surg. Case Rep.*, vol. 41, pp. 110–113, Oct. 2017, doi: 10.1016/j.ijscr.2017.10.024.
[36] I. Kahramanoglu, V. Sal, and T. Bese, “Post-coital vaginal cuff dehiscence with small bowel evisceration after laparoscopic type II radical hysterectomy: A case report,” *Int. J. Surg. Case Rep.*, vol. 26, pp. 81–83, 2016, doi: 10.1016/j.ijscr.2016.07.024.
[37] S. Kalogeropoulos, E. Chronopoulou, E. Iliopoulos, and G. Decavalas, “Vaginal cuff dehiscence after laparoscopic trachelectomy post hysterectomy: a case report and brief review of the literature,” *Int. J. Reprod. Contracept. Obstet. Gynecol.*, vol. 7, no. 12, pp. 5204–5206, Nov. 2018, doi: 10.18203/2320-1770.ijrcog20184996.
[38] A. A. Kambouris, B. H. Drukker, and J. Barron, “Vaginal evisceration. A case report and brief review of the literature,” *Arch. Surg. Chic. Ill 1960*, vol. 116, no. 7, pp. 949–951, Jul. 1981, doi: 10.1001/archsurg.1981.01380190077018.
[39] W. D. Kang, S. M. Kim, and H. S. Choi, “Vaginal evisceration after radical hysterectomy and adjuvant radiation,” *J. Gynecol. Oncol.*, vol. 20, no. 1, pp. 63–64, Mar. 2009, doi: 10.3802/jgo.2009.20.1.63.
[40] S.-M. Kim, H.-S. Choi, J.-S. Byun, Y.-S. Kim, and H.-R. Kim, “Transvaginal evisceration after radical abdominal hysterectomy,” *Gynecol. Oncol.*, vol. 85, no. 3, pp. 543–544, Jun. 2002, doi: 10.1006/gyno.2002.6658.
[41] Y. Kim, M. C. Morris, and R. E. Earnest, “Surgical management of post-traumatic transvaginal herniation of small intestine in a third-world country,” *BMJ Case Rep.*, vol. 12, no. 5, p. e228330, May 2019, doi: 10.1136/bcr-2018-228330.
[42] I. Kızıloglu, S. Karaıslı, E. Sarı, M. Bayındır, and E. Tarcan, “A Rare general surgical emergency : small bowel evisceration from vaginal cuff six years after hysterectomy,” *Acta Gastro-Enterol. Belg.*, vol. 80, no. 3, pp. 427–428, Sep. 2017.
[43] S. Kumar, “Recurrent Vaginal Cuff Dehiscence in a Treated Case of Carcinoma Cervix,” *J. Clin. Diagn. Res.*, 2017, doi: 10.7860/JCDR/2017/28389.10508.
[44] Y.-P. Lan, H.-H. Chen, W.-M. Liu, and C.-H. Chen, “Delayed postcoital vaginal cuff dehiscence with small bowel evisceration after robotic-assisted staging surgery,” *Taiwan. J. Obstet. Gynecol.*, vol. 56, no. 2, pp. 258–260, Apr. 2017, doi: 10.1016/j.tjog.2016.02.022.
[45] L. Lawson, L. Patterson, and K. Carter, “Transvaginal evisceration progressing to peritonitis in the emergency department: a case report,” *Int. J. Emerg. Med.*, vol. 4, no. 1, p. 66, Oct. 2011, doi: 10.1186/1865-1380-4-66.
[46] D. H. Lee *et al.*, “Spontaneous reduction of transvaginal small bowel evisceration after abdominal hysterectomy for cervical cancer: A case report,” *Medicine (Baltimore)*, vol. 101, no. 17, p. e29225, Apr. 2022, doi: 10.1097/MD.0000000000029225.
[47] F. C. Lin, A. Medendorp, M. Van Kuiken, S. A. Mills, and C. M. Tarnay, “Vaginal Dehiscence and Evisceration After Robotic-assisted Radical Cystectomy: A Case Series and Review of the Literature,” *Urology*, vol. 134, pp. 90–96, Dec. 2019, doi: 10.1016/j.urology.2019.09.009.

[48] J. B. Lledó, M. P. Roig, A. S. Serra, C. R. Astaburuaga, and F. D. Giménez, “Laparoscopic repair of vaginal evisceration: a case report,” *Surg. Laparosc. Endosc. Percutan. Tech.*, vol. 12, no. 6, pp. 446–448, Dec. 2002, doi: 10.1097/00129689-200212000-00013.
[49] R. Manchanda and A. Refaie, “Acute pneumoperitoneum following coitus,” *CJEM*, vol. 7, no. 1, pp. 51–53, Jan. 2005, doi: 10.1017/s148180350001294x.
[50] S. A. Mastrolia *et al.*, “Vaginal Treatment of Vaginal Cuff Dehiscence with Visceral Loop Prolapse: A New Challenge in Reparative Vaginal Surgery?,” *Case Rep. Obstet. Gynecol.*, vol. 2014, p. 257398, 2014, doi: 10.1155/2014/257398.
[51] T. Matsuhashi, K. Nakanishi, E. Hamano, S. Kamoi, and T. Takeshita, “Laparoscopic Repair of Vaginal Evisceration after Abdominal Hysterectomy for Uterine Corpus Cancer: A Case Report and Literature Review,” *J. Nippon Med. Sch. Nippon Ika Daigaku Zasshi*, vol. 84, no. 2, pp. 90–95, 2017, doi: 10.1272/jnms.84.90.
[52] M. C. McCullough and S. Hart, “Laparoscopic repair of postcoital vaginal evisceration with intact pelvic organs,” *JSLS*, vol. 16, no. 1, pp. 182–183, Mar. 2012, doi: 10.4293/108680812X13291597716825.
[53] B. C. McMaster and C. Molins, “Small Bowel Evisceration after Spontaneous Vaginal Cuff Rupture,” *Cureus*, vol. 11, no. 8, p. e5535, Aug. 2019, doi: 10.7759/cureus.5535.
[54] J. A. Mínguez, M. Auba, and D. Diaz-Cobos, “Vaginal evisceration in a patient with previous sacrocolpopexy,” *Int. Urogynecology J.*, vol. 22, no. 12, pp. 1597–1599, Dec. 2011, doi: 10.1007/s00192-011-1463-7.
[55] G. Missori, N. L. A. Marchesini, D. Mosca, A. A. Ricciardolo, F. Serra, and R. Gelmini, “Vaginal Evisceration of Small Bowel With Extraperitoneal Ileal Resection of the Herniated Loops: A Case Report,” *Front. Surg.*, vol. 9, p. 878760, 2022, doi: 10.3389/fsurg.2022.878760.
[56] N. Mohamed Salad, A. Ali Omar, and Y. G. Mohamed, “Spontaneous transvaginal small bowel evisceration secondary to vaginal cuff dehiscence after abdominal hysterectomy: A case report,” *Ann. Med. Surg. 2012*, vol. 79, p. 103986, Jul. 2022, doi: 10.1016/j.amsu.2022.103986.
[57] M. Muraoka, H. Nagano, and K. Takagi, “Evisceration occurred 1 year after vaginal vault repair for relapsed pelvic organ prolapse,” *J. Obstet. Gynaecol. Res.*, vol. 38, no. 7, pp. 1028–1031, Jul. 2012, doi: 10.1111/j.1447-0756.2011.01825.x.
[58] F. Narducci, Y. Sonoda, E. Lambaudie, E. Leblanc, and D. Querleu, “Vaginal evisceration after hysterectomy: the repair by a laparoscopic and vaginal approach with a omental flap,” *Gynecol. Oncol.*, vol. 89, no. 3, pp. 549–551, Jun. 2003, doi: 10.1016/S0090-8258(03)00153-7.
[59] A. O. Nasr, S. Tormey, M. A. Aziz, and B. Lane, “Vaginal herniation: case report and review of the literature,” *Am. J. Obstet. Gynecol.*, vol. 193, no. 1, pp. 95–97, Jul. 2005, doi: 10.1016/j.ajog.2004.12.022.
[60] J. R. Negrete *et al.*, “Transvaginal evisceration of the small bowel a rare and potentially lethal event, a case report,” *Ann. Med. Surg.*, vol. 65, p. 102352, May 2021, doi: 10.1016/j.amsu.2021.102352.
[61] C. F. Newell, N. P. Larson, and M. J. Yoo, “Small Bowel in Vagina: A Case for Pelvic Exams,” *Cureus*, vol. 13, no. 8, p. e17412, Aug. 2021, doi: 10.7759/cureus.17412.
[62] C. H. Nezhat, F. Nezhat, D. S. Seidman, and C. Nezhat, “Vaginal vault evisceration after total laparoscopic hysterectomy,” *Obstet. Gynecol.*, vol. 87, no. 5 Pt 2, pp. 868–870, May 1996.
[63] M.-L. T. Nguyen, A. L. Anyikam, and M. Paolucci, “Vaginal cuff dehiscence with adnexal mass evisceration after abdominal hysterectomy,” *Int. J. Surg. Case Rep.*, vol. 4, no. 5, pp. 518–520, 2013, doi: 10.1016/j.ijscr.2013.02.012.
[64] M.-L. T. Nguyen, M. Kapoor, T. S. Pradhan, T. L. Pua, and S. S. Tedjarati, “Two cases of post-coital vaginal cuff dehiscence with small bowel evisceration after robotic-assisted laparoscopic hysterectomy,” *Int. J. Surg. Case Rep.*, vol. 4, no. 7, pp. 603–605, 2013, doi: 10.1016/j.ijscr.2013.04.001.
[65] I. Nikolopoulos, H. Khan, G. Janakan, and R. Kerwat, “Laparoscopically assisted repair of vaginal evisceration after hysterectomy,” *BMJ Case Rep.*, vol. 2013, p. bcr2013009897, May 2013, doi: 10.1136/bcr-2013-009897.
[66] S. Orito, N. Masuya, A. Sakurabashi, and S. Minoura, “Vaginal evisceration 3 years after abdominal hysterectomy and bilateral salpingo-oophorectomy,” *J. Obstet. Gynaecol. Res.*, vol. 38, no. 12, pp. 1385–1388, Dec. 2012, doi: 10.1111/j.1447-0756.2012.01877.x.
[67] R. S. Parra, J. J. R. da Rocha, and O. Feres, “Spontaneous transvaginal small bowel evisceration: a case report,” *Clin. Sao Paulo Braz.*, vol. 65, no. 5, pp. 559–561, May 2010, doi: 10.1590/S1807-59322010000500015.
[68] G. A. Partsinevelos, A. Rodolakis, S. Athanasiou, and A. Antsaklis, “Vaginal evisceration after hysterectomy: a rare condition a gynecologist should be familiar with,” *Arch. Gynecol. Obstet.*, vol. 279, no. 2, pp. 267–270, Feb. 2009, doi: 10.1007/s00404-008-0820-3.
[69] F. A. Pereira and H. Rai, “A rare case of vaginal vault evisceration and its management,” *J. Surg. Case Rep.*, vol. 2012, no. 5, p. 6, May 2012, doi: 10.1093/jscr/2012.5.6.
[70] C. J. Pinto, S. Hegde, S. Karekal, D. Anneaud, and A. Sharma, “Spontaneous Transvaginal Small Bowel Evisceration With Uterine Procidentia: A Report of a Rare Case,” *Cureus*, vol. 14, no. 7, p. e27319, Jul. 2022, doi: 10.7759/cureus.27319.
[71] S. Rajesh, E. Kalu, J. Bong, and N. Wales, “Evisceration 5 years post abdominal hysterectomy,” *J. Obstet. Gynaecol. Res.*, vol. 34, no. 3, pp. 425–427, Jun. 2008, doi: 10.1111/j.1447-0756.2008.00778.x.
[72] A. M. Rana, A. A. Rana, and Y. Salama, “Small Bowel Evisceration through the Vaginal Vault: A Rare Surgical Emergency,” *Cureus*, vol. 11, no. 10, p. e5947, Oct. 2019, doi: 10.7759/cureus.5947.

[73] S. P. Ribeiro, A. C. Silva, J. Maciel, and A. S. Antunes, “Spontaneous transvaginal evisceration: a case of recurrence,” *BMJ Case Rep.*, vol. 2016, p. bcr2015211659, Feb. 2016, doi: 10.1136/bcr-2015-211659.
[74] B. L. Robinson, J. B. Liao, S. F. Adams, and T. C. Randall, “Vaginal cuff dehiscence after robotic total laparoscopic hysterectomy,” *Obstet. Gynecol.*, vol. 114, no. 2 Pt 1, pp. 369–371, Aug. 2009, doi: 10.1097/AOG.0b013e3181af68c6.
[75] P. Rogers, H. Lee, K. Jape, Z. Q. Ng, and D. Koong, “Vaginal evisceration of small bowel,” *J. Surg. Case Rep.*, vol. 2019, no. 11, p. rjz317, Nov. 2019, doi: 10.1093/jscr/rjz317.
[76] A. M. Schefter, J. Fischer, and B. K. Erickson, “Spontaneous Transvaginal Sigmoid Colon Evisceration and Sepsis From Complete Procidentia,” *Female Pelvic Med. Reconstr. Surg.*, vol. 24, no. 6, pp. e42–e45, Dec. 2018, doi: 10.1097/SPV.0000000000000611.
[77] L. Schreiner, T. G. Santos, C. C. Nygaard, and D. S. Oliveira, “Vaginal evisceration related to genital prolapse in premenopausal woman,” *Int. Braz J Urol Off. J. Braz. Soc. Urol.*, vol. 43, no. 4, pp. 766–769, Aug. 2017, doi: 10.1590/S1677-5538.IBJU.2016.0249.
[78] F. Sendy, L. D. Simone, M. Albaut, A. Lambert, and E. Nohuz, “Laparoscopic assessment and transvaginal reparation of post-coital vaginal cuff dehiscence with bowel evisceration: a case report,” *Pan Afr. Med. J.*, vol. 35, Apr. 2020, doi: 10.11604/pamj.2020.35.118.2136.
[79] F. Shehata, O. Bougie, K. Baker, and H. Khalil, “Occult Vaginal Evisceration: A Case Report,” *J. Obstet. Gynaecol. Can. JOGC J. Obstet. Gynecol. Can. JOGC*, vol. 41, no. 9, pp. 1341–1343, Sep. 2019, doi: 10.1016/j.jogc.2018.11.025.
[80] I. Siddiqui, A. Samee, C. Hall, J. Cooper, and F. O’Mahony, “Spontaneous vaginal evisceration,” *BMJ Case Rep.*, vol. 2011, p. bcr1020103410, Mar. 2011, doi: 10.1136/bcr.10.2010.3410.
[81] M. D. J. Sinclair, A. R. Davies, S. Sankaran, S. Agnihotri, and S. M. Andreani, “Laparoscopic repair of spontaneous vaginal evisceration of small bowel: report of a case,” *Ann. R. Coll. Surg. Engl.*, vol. 92, no. 1, pp. W3-5, Jan. 2010, doi: 10.1308/147870810X476610.
[82] E. R. Sokol and E. G. Munro, “Incarcerated vaginal herniation of the omentum mimicking vaginal prolapse,” *Am. J. Obstet. Gynecol.*, vol. 196, no. 3, pp. e7-8, Mar. 2007, doi: 10.1016/j.ajog.2006.11.030.
[83] J. Solberg and K. Saravana, “Omental Prolapse Through Vaginal Cuff Dehiscence,” *Clin. Pract. Cases Emerg. Med.*, vol. 6, no. 3, pp. 262–263, Aug. 2022, doi: 10.5811/cpcem.2022.2.56353.
[84] S. G. Somkuti, P. A. Vieta, J. F. Daugherty, L. W. Hartley, and E. B. Blackmon, “Transvaginal evisceration after hysterectomy in premenopausal women: a presentation of three cases,” *Am. J. Obstet. Gynecol.*, vol. 171, no. 2, pp. 567–568, Aug. 1994, doi: 10.1016/0002-9378(94)90306-9.
[85] G. Stabile *et al.*, “Postcoital Vaginal Perforation and Evisceration in Women with No Prior Pelvic Surgery: Laparoscopic Management and Systematic Review of the Literature,” *Int. J. Environ. Res. Public. Health*, vol. 18, no. 18, p. 9746, Sep. 2021, doi: 10.3390/ijerph18189746.
[86] E. Sterk and K. Stonewall, “Vaginal cuff dehiscence – A potential surgical emergency,” *Am. J. Emerg. Med.*, vol. 38, no. 3, p. 691.e1-691.e2, Mar. 2020, doi: 10.1016/j.ajem.2019.09.013.
[87] P. Szymczak and D. G. Wydra, “Evisceration of the small intestine through the vagina as a rare complication after laparoscopic pectopexy,” *Ginekol. Pol.*, Sep. 2021, doi: 10.5603/GP.a2021.0163.
[88] T. Thomopoulos and G. Zufferey, “Totally laparoscopic treatment of vaginal cuff dehiscence: A case report and systematic literature review,” *Int. J. Surg. Case Rep.*, vol. 25, pp. 79–82, Jan. 2016, doi: 10.1016/j.ijscr.2016.06.004.
[89] J. W. T. Toh *et al.*, “Transvaginal evisceration of small bowel,” *ANZ J. Surg.*, vol. 89, no. 6, pp. 774–776, Jun. 2019, doi: 10.1111/ans.14290.
[90] B. Vardar and B. Midkiff, “Vaginal cuff dehiscence: report of two cases,” *Radiol. Case Rep.*, vol. 16, no. 8, pp. 2231–2235, Jun. 2021, doi: 10.1016/j.radcr.2021.05.038.
[91] C. A. Walsh, J. R. A. Sherwin, and M. Slack, “Vaginal evisceration following total laparoscopic hysterectomy: case report and review of the literature,” *Aust. N. Z. J. Obstet. Gynaecol.*, vol. 47, no. 6, pp. 516–519, Dec. 2007, doi: 10.1111/j.1479-828X.2007.00793.x.
[92] W. A. Wan Hassan, V. Narasimhan, A. Arachchi, T. Manolitsas, and W. Teoh, “Small bowel evisceration from vagina,” *J. Surg. Case Rep.*, vol. 2021, no. 8, p. rjab343, Aug. 2021, doi: 10.1093/jscr/rjab343.
[93] K. C. Woo, J. A. Linden, R. A. Lowenstein, J. C. Varghese, and M. A. Burch, “Subtle vaginal evisceration resulting in small bowel evisceration: a case report,” *J. Emerg. Med.*, vol. 43, no. 2, pp. e125-128, Aug. 2012, doi: 10.1016/j.jemermed.2011.06.003.
[94] M. D. Yaakovian, G. G. Hamad, and R. S. Guido, “Laparoscopic management of vaginal evisceration: case report and review of the literature,” *J. Minim. Invasive Gynecol.*, vol. 15, no. 1, pp. 119–121, Feb. 2008, doi: 10.1016/j.jmig.2007.08.618.
[95] F. Yanar, G. Oner, B. Ozcinar, A. F. K. Gok, and C. Ertekin, “Spontaneous transvaginal small bowel evisceration following hysterectomy: a case report,” *Ulus. Travma Ve Acil Cerrahi Derg. Turk. J. Trauma Emerg. Surg. TJTES*, vol. 25, no. 4, pp. 424–426, Jul. 2019, doi: 10.14744/tjtes.2019.57318.
[96] K. Yüce, P. Dursun, and M. Gültekin, “Posthysterectomy intestinal prolapse after coitus and vaginal repair,” *Arch. Gynecol. Obstet.*, vol. 272, no. 1, pp. 80–81, Jun. 2005, doi: 10.1007/s00404-004-0709-8.
[97] Y. Zhou *et al.*, “Spontaneous vaginal cuff dehiscence and evisceration of multiple organs: A case report,” *Medicine (Baltimore)*, vol. 97, no. 50, p. e13670, Dec. 2018, doi: 10.1097/MD.0000000000013670.
